# Autochthonous Dengue Fever, Tokyo, Japan, 2014

**DOI:** 10.3201/eid2103.141662

**Published:** 2015-03

**Authors:** Satoshi Kutsuna, Yasuyuki Kato, Meng Ling Moi, Akira Kotaki, Masayuki Ota, Koh Shinohara, Tetsuro Kobayashi, Kei Yamamoto, Yoshihiro Fujiya, Momoko Mawatari, Tastuya Sato, Junwa Kunimatsu, Nozomi Takeshita, Kayoko Hayakawa, Shuzo Kanagawa, Tomohiko Takasaki, Norio Ohmagari

**Affiliations:** National Center for Global Health and Medicine, Tokyo, Japan (S. Kutsuna, Y. Kato, M. Ota, K. Shinohara, T. Kobayashi, K. Yamamoto, Y. Fujiya, M. Mawatari, T. Sato, J. Kunimatsu, N. Takeshita, K. Hayakawa, S. Kanagawa, N. Ohmagari); National Institute of Infectious Diseases, Tokyo (M.L. Moi, A. Kotaki, T. Takasaki)

**Keywords:** Dengue fever, Aedes albopictus, Tokyo, Japan, parasites, viruses, mosquitoes

## Abstract

After 70 years with no confirmed autochthonous cases of dengue fever in Japan, 19 cases were reported during August–September 2014. Dengue virus serotype 1 was detected in 18 patients. Phylogenetic analysis of the envelope protein genome sequence from 3 patients revealed 100% identity with the strain from the first patient (2014) in Japan.

Although ≈200 imported cases of dengue fever have recently been reported in Japan (*1*), an autochthonous case had not been confirmed there for 70 years (*2*). However, on August 26, 2014, an autochthonous case of dengue fever in a patient with no history of overseas travel was reported in Tokyo, and as of October 31, 2014, a total of 160 autochthonous cases in Japan had been confirmed (*3*).

## The Cases

We report 19 cases of confirmed autochthonous dengue fever treated at the National Center for Global Health and Medicine in Tokyo, Japan, during August 26–September 22, 2014 (Tables 1, 2; Technical Appendix Table). Because the National Center for Global Health and Medicine is located close to the epicenter of this outbreak, 19 (12%) of the 160 cases of this outbreak were confirmed at this Center. Informed consent for participation in this study was obtained from all 19 patients. Of these 19 patients, the median age was 33.0 years (range 6–64 years), and 10 (55.6%) were men. None of the patients had any underlying illness except for hypertension (2 patients) and asthma (1 patient). One patient had a history of having contracted dengue fever while in the Philippines in 2006. None of the patients had traveled overseas during the 3 months before the outbreak of dengue virus type 1 (DENV-1) in Japan. 

**Table 1:** Clinical characteristics of 19 patients with dengue fever, Tokyo, Japan, August 26, 2014–September 22, 2014

Sign or symptom	Patients, no. (%)
Fever*	19 (100)
Headache	17 (89.5)
Arthralgia	7 (36.8)
Myalgia	7 (36.8)
Nausea	5 (26.3)
Vomiting	2 (10.5)
Diarrhea	1 (5.3)
Rash at first visit	4 (21.1)
Rash during course of illness	15 (78.9)
Sore throat	2 (10.5)
Cough	4 (21.1)
Sputum	1 (5.3)

*Median duration of fever >38°C = 7 (range 4–11) days.

**Table 2:** Laboratory findings for 19 patients with dengue fever, Tokyo, Japan, August 26, 2014–September 22, 2014

Laboratory finding at first visit	Reference range	Patient median (interquartile range)
Leukocytes, cells /mm³	3,500-8,500	2,600 (2,385–3,540)
Hematocrit, %	M 40–50, F 35–45	41.8 (38.5–42.9)
Platelets, ×10^3^/μL	150–350	115 (79–150)
Aspartate transaminase, IU/L	13–33	35 (22–41)
Alanine transaminase, IU/L	8–42	20 (14–26)
Lactate dehydrogenase, IU/L	119–229	227 (166–261)
C-reactive protein, mg/L	0–0.3	6.1 (2.2–16.1)

**Figure 1:**
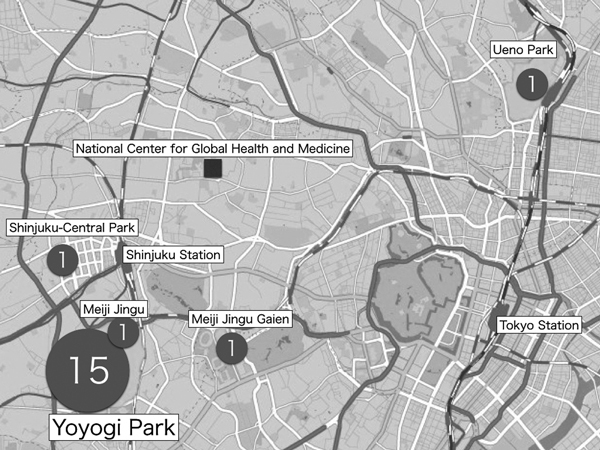
Locations of presumptive exposure to dengue virus mosquito vectors for 19 patients, Tokyo, Japan, August 26–September 22, 2014. Numbers in circles indicate numbers of cases contracted at each location.

Places of exposures were assessed for all patients; 15 patients had recently visited Yoyogi Park and were bitten by mosquitoes while there; the remaining 4 patients had visited Shinjuku Central Park, Meiji Jingu Shrine, Meijiingu Gaien, and Ueno Park. All of these parks have been reported as affected regions in this outbreak (*3*) (Figure 1). The day of exposure was estimated for 9 patients for whom the day of visitation and mosquito bites while in the parks could be confirmed. Among these 9 patients, the median incubation period was 6 (range 3–9) days. For the other 10 patients, the incubation period was not determined because they had visited the parks over several days or because they lived near these parks. The dates of symptom onset ranged from August 12, 2014, through September 22, 2014; peak incidence occurred in the beginning of September. 

Of the 19 patients, 16 were admitted to the National Center for Global Health and Medicine and discharged without sequelae; the other 3 received outpatient treatment and recovered. The patient with a history of dengue fever (patient 19 in the Technical Appendix Table) experienced fever lasting 7 days, pleural effusion, spontaneous petechiae, and thrombocytopenia (15 × 10^3^ cells/μL on day 8 after illness onset); dengue hemorrhagic fever was diagnosed for this patient by using the World Health Organization guidelines (*4*). On the day of illness onset for this patient, serum was positive for DENV nonstructural protein 1 (NS1) antigen and IgG but negative for DENV IgM. These results demonstrated that this DENV infection was secondary. Epidemiologic studies have also shown that the risk for dengue hemorrhagic fever is significantly higher for patients with secondary rather than primary DENV infection (*5*).

**Figure 2:**
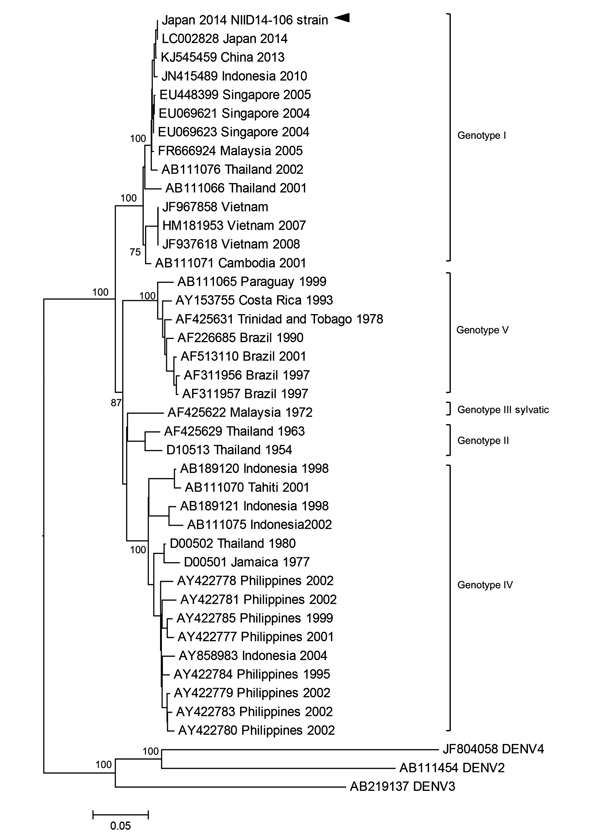
Phylogenetic analysis of a dengue virus (DENV) sequence derived from a patient with confirmed autochthonous dengue fever (patient 2), Tokyo, Japan, contracted during August 26–September 22, 2014. Phylogenetic tree is based on the envelope protein genome sequence of selected dengue virus type-1 (DENV-1) strains. DENV-2, DENV-3, and DENV-4 serotypes were used as outgroups. Percentages of successful bootstrap replication are indicated at the nodes. DENV-1 genotypes are indicated on the right. The DENV-1 National Institute of Infectious Diseases (NIID) strain 14-106 (GenBank accession no. LC006123) is indicated with an arrowhead. Virus strains are indicated by GenBank accession number, place, and date of isolation. Scale bar indicates number of nucleotide substitutions per site.

Of the 19 cases, 18 were confirmed as DENV-1 infection by real-time PCR (TaqMan; Life Technologies, Grand Island, NY, USA) (*6*), and samples were positive for NS1 antigen (Platelia Dengue NS1 Antigen assay; Bio-Rad Laboratories, Marnes-la-Coquette, France). The remaining case (case 11) was confirmed positive for IgM and IgG against DENV by dengue IgM ELISA (Focus Diagnostics, Inc., Cypress, CA, USA) and dengue IgG ELISA (Vircell, Granada, Spain), respectively. The serotype of the DENV in the other 18 patients was confirmed to be serotype 1. Nucleotide sequences were determined by using BigDye Terminator version 3.1 (Applied Biosystems, Foster City, CA, USA). Phylogenetic analysis of the DENV envelope (E) protein genome sequence obtained from serum from patient 2 (GenBank accession no. LC006123) demonstrated that the E protein shared 100% homology with the sequence of a DENV-1 strain from the first patient in this outbreak in Japan (GenBank accession no. LC002828). The sequence from patient 2 shared 99.7% identity with the sequence of a DENV strain isolated in Guangzhou, China, in 2013 (GenBank accession no. KJ545459) and 99.3% identity with the sequence of a DENV strain isolated in Indonesia in 2010 (GenBank accession no. JN415489) (Figure 2). The sequence of the E protein from another 2 patients (patients 4 and 10) shared 100% homology with that of patient 2.

## Conclusions

Our results suggest that a single strain may have caused most of the DENV cases in Tokyo. A similar outbreak of dengue fever had been reported in Ningbo, China (68 cases) (*7*), and in Hawaii, USA (122 cases) (*8*). 

DENV is transmitted mainly through the bite of the *Aedes aegypti* mosquito, which is distributed in tropical and subtropical regions. In Japan, the distribution of *Ae. aegypti* mosquitoes is limited, and as of 2013, these mosquitoes had been found only at the Narita International Airport (*9*), which is located ≈60 km from the site of the DENV outbreak in Tokyo. In contrast, the distribution of *Ae. Albopictus* mosquitoes*,* another vector of DENV, extends from western regions to northern regions of Japan, including Tokyo. *Ae. albopictus* mosquitoes are also expanding into the northern regions of the main island because of global warming (*10*). Tokyo is one of the most heavily populated cities in the world, and Yoyogi Park is located at the center of the Shinjuku-Shibuya area in Tokyo.

The population density of *Ae. albopictus* mosquitoes in Tokyo is higher than that in suburban areas (*11*). It is possible that high human and mosquito population densities contributed to this outbreak. All 19 patients had been bitten by a mosquito while in Tokyo, mainly in Yoyogi Park, where most of the 160 patients with autochthonous dengue cases had also been (*12*). 

Recently, a case of dengue fever imported to England from Japan was found to be associated with this outbreak (*13*). Previous investigators speculated that the virus may have been spread from infected visitors by mosquitoes in the park (*13*). The outbreak, however, coincides with a period during which several tropical-themed festivals and activities were hosted, July–August 2014. These activities attracted a high number of local and international visitors to the park.

Before this 2014 outbreak, dengue fever was diagnosed for a German traveler who had returned from Japan in 2013 (*14*). Neutralization tests confirmed that the traveler’s infection was caused by DENV-2. She was reportedly bitten by mosquitoes when in Fuefuki, but she had also traveled to Tokyo and Kyoto during her trip to Japan. No local DENV cases were reported in 2013, although cases may have been underreported because of the lack of local dengue outbreaks in Japan for the past 70 years. 

Because adult *Ae. albopictus* mosquitoes cannot survive the winter in Japan, only eggs overwinter (*15*). Thus, the outbreak of autochthonous dengue fever is expected to end as mosquito activity decreases during autumn. The Japanese government is currently strengthening vector control measures and increasing awareness among residents to prevent similar outbreaks.

Technical Appendix Characteristics of 19 patients with dengue fever, Tokyo, Japan, August 26, 2014–September 22, 2014.
