# Supplementary material for: Autochthonous Dengue Fever, Tokyo, Japan, 2014
Source: Emerg Infect Dis. 2015 Mar;21(3):517–20. doi: 10.3201/eid2103.141662 (PMC4344289; doi:10.3201/eid2103.141662)
Supplement: Technical Appendix — Characteristics of 19 patients with dengue fever, Tokyo, Japan, August 26, 2014–September 22, 2014. [file 14-1662-Techapp-s1.pdf]

Article DOI: http://dx.doi.org/10.3201/eid2103.141662

Dengue Fever, Tokyo, Japan, 2014

Technical Appendix

Appendix Table. Clinical and laboratory values for 19 patients with dengue fever, Tokyo, Japan, August 26, 2014–September 22, 2014

											Clinical Symptoms													Laboratory Findings at the first presentation							ELISA			
Case	age	sex	underlying illness / past history	date of first presentation to NCGM	date of onset	days from onset to first presentation	date of exposure	estimated incubation period	presumptive exposure place	travel history of overseas	fever	duration of fever above 38℃ (days)	headache	arthralgia	myalgia	nausea	vomit	diarrhea	rash at the first presentation	rash during the course	sore throat	cough	sputum	WBC (/mm³)	Ht (%)	Plt ($\times 10^4$/μL)	AST (IU/L)	ALT (IU/L)	LDH (IU/L)	CRP (mg/dl)	NS1	IgM	IgG	RT-PCR (RNA copies/ml)
1	16	M	-	8/26	8/18	8	unknown	NA	Yoyogi Park	-	+	9	+	-	-	+	+	-	+	+	+	+	+	2600	44.5	9.2	65	186	330	0.50	7.9	7.0	2.7	DENV-1 (1.7E5)
2	23	M	-	8/27	8/24	3	unknown	NA	Yoyogi Park	-	+	6	+	+	+	+	-	-	-	-	+	-	-	2530	50.1	11.5	27	16	224	0.84	8.0	0.61	0.55	DENV-1 (5.1E8)
3	34	F	-	8/29	8/23	6	8/16	8	Yoyogi Park	-	+	7	-	-	-	+	-	-	-	+	-	-	-	1190	41.6	7.0	29	23	230	0.04	17	2.5	1.7	DENV-1 (2.9E5)
4	56	M	-	8/29	8/25	4	unknown	NA	Yoyogi Park	-	+	6	+	-	-	-	-	-	-	+	-	-	-	1200	41.7	8.8	42	28	236	0.15	17	1.5	1.6	DENV-1 (3.1E8)
5	20	M	-	8/29	8/25	4	8/19-20	NA	Yoyogi Park	-	+	7	+	-	+	-	-	-	-	+	-	-	-	2030	42.1	11.1	38	22	NA	0.61	7.9	2.5	1.8	DENV-1 (1.6E6)
6	48	F	-	8/29	8/29	0	unknown	NA	Yoyogi Park	-	+	6	+	+	+	-	-	-	-	+	-	-	-	5470	38.3	14.4	20	14	NA	0.12	17	0.45	0.28	DENV-1 (3.1E8)
7	55	M	HT	9/1	8/30	2	unknown	NA	Yoyogi Park	-	+	9	+	+	+	-	-	-	+	+	-	-	-	3100	42.7	11.3	50	48	216	3.35	8.0	0.72	0.18	DENV-1 (1.2E10)
8	42	F	-	9/1	8/31	1	unknown	NA	Yoyogi Park	-	+	6	+	+	-	-	-	-	-	-	-	-	-	3590	36.6	13.7	20	13	210	3.37	8.0	0.75	0.20	DENV-1 (1.9E9)
9	28	F	-	9/2	9/1	1	8/28	4	Yoyogi Park	-	+	7	+	+	+	-	-	-	-	-	-	+	-	5550	37.5	17.9	23	17	144	2.37	7.9	0.49	0.58	DENV-1 (9.6E8)
10	64	M	HT	9/4	9/1	3	8/29	3	Meijijingu Gaien	-	+	7	+	+	-	-	-	-	-	+	-	-	-	2580	41.8	15.5	37	23	NA	1.93	9.3	0.61	0.23	DENV-1 (3.0E9)
11	46	M	-	9/4	8/12	24	8/7-10	NA	Yoyogi Park	-	+	NA	+	-	-	-	-	-	-	+	-	-	-	3630	50.7	6.3	126	82	466	0.17	0.18	12	2.8	negative
12	6	M	asthma	9/5	9/1	4	8/26	6	Yoyogi Park	-	+	5	-	-	-	-	-	-	-	+	-	+	-	2600	36.8	22.0	35	12	233	1.28	not examined	not examined	not examined	DENV-1
13	20	F	-	9/6	9/5	1	8/27	9	Yoyogi Park	-	+	11	+	-	+	-	-	-	-	+	-	-	-	3490	34.4	15.9	19	10	153	1.13	8.0	0.53	0.47	DENV-1 (4.2E8)
14	51	F	-	9/6	9/5	1	unknown	NA	Yoyogi Park	-	+	7	+	-	+	-	-	-	-	+	-	-	-	3120	38.7	14.2	25	14	165	3.50	7.9	0.69	0.14	DENV-1 (1.9E9)
15	28	F	-	9/9	9/8	1	9/2	6	Meiji Jingu Shine	-	+	6	+	-	-	-	-	-	-	+	-	-	-	2450	42.4	15.6	18	10	118	1.04	7.9	0.58	0.25	DENV-1 (2.2E9)
16	33	M	-	9/11	9/5	6	9/2	3	Yoyogi Park	-	+	4	+	+	-	-	-	-	+	+	-	-	-	2400	43.9	5.7	85	92	368	0.27	8.0	3.9	0.94	DENV-1 (5.1E4)
17	23	F	-	9/14	9/7	7	unknown	NA	Shinjuku Central Park	-	+	7	+	-	-	+	+	-	+	+	-	-	-	2370	44.0	4.2	201	103	647	0.26	8.0	4.3	2.6	DENV-1 (5.0E6)
18	23	F	-	9/17	9/13	4	9/7	6	Ueno Park	-	+	7	+	-	-	+	-	-	-	+	-	-	-	1450	41.6	6.5	40	20	337	0.14	13	2.4	1.2	DENV-1

Case	age	sex	underlying illness / past history	date of first presentation to NCGM	date of onset	days from onset to first presentation	date of exposure	estimated incubation period	presumptive exposure place	travel history of overseas	Clinical Symptoms													Laboratory Findings at the first presentation							ELISA			RT-PCR (RNA copies/ml)
											fever	duration of fever above 38℃ (days)	headache	arthralgia	myalgia	nausea	vomit	diarrhea	rash at the first presentation	rash during the course	sore throat	cough	sputum	WBC ($/mm^3$)	Ht (%)	Plt ($\times 10^4/\mu L$)	AST (IU/L)	ALT (IU/L)	LDH (IU/L)	CRP (mg/dl)	NS1	IgM	IgG	
																																		(6.1E5)
19	44	M	dengue fever	9/19	9/13	7	9/4	9	Yoyogi Park	-	+	7	+	-	-	-	-	+	-	-	-	+	-	4000	43.1	14.5	19	12	166	0.27	6.7	0.9	2.7	DENV-1

M: Male, F: Female, NA: Not Available, HT: Hypertension, DENV: Dengue Virus, WBC: White Blood Cells(standard value; 3500-8500/mm^3), Ht: Hematocrit(Male: 40-50%, Female 35-45%), Plt: Platelet(150-350$\times 10^9$/L), AST: Aspartate Transaminase(13-33 IU/L), ALT: Alanine Transaminase(8-42 IU/L), LDH: Lactate Dehydrogenase(119-229 IU/L), CRP: C-reactive Protein(0-0.3 mg/dl), ELISA: Enzyme-Linked ImmunoSorbent Assay, NS1: The Non-Structural Protein 1 Antigen, IgM: Immunoglobulin M, IgG: Immunoglobulin G, RT-PCR: Real-Time Polymerase Chain Reaction
Abnormal values are bolded.
